# Gut microbiota and gastrointestinal tumors: insights from a bibliometric analysis

**DOI:** 10.3389/fmicb.2025.1558490

**Published:** 2025-04-08

**Authors:** Chaofan Chen, Xiaolan Wang, Xu Han, Lifan Peng, Zhiyun Zhang

**Affiliations:** ^1^Department of Anorectal, Kunming Municipal Hospital of Traditional Chinese Medicine, The Third Affiliated Hospital of Yunnan University of Chinese Medicine, Kunming, Yunnan, China; ^2^Department of Anorectal, Kunming Municipal Hospital of Traditional Chinese Medicine, The Third Affiliated Hospital of Yunnan University of Chinese Medicine, Kunming, Yunnan, China

**Keywords:** CiteSpace, VOSviewer, gut microbiota, emerging research trends, gastrointestinal tumors, immunotherapy

## Abstract

**Introduction:**

Despite the growing number of studies on the role of gut microbiota in treating gastrointestinal tumors, the overall research trends in this field remain inadequately characterized.

**Methods:**

A bibliometric analysis was conducted using publications retrieved from the Web of Science Core Collection (up to September 30, 2024). Analytical tools including VOSviewer, CiteSpace, and an online bibliometric platform were employed to evaluate trends and hotspots.

**Results:**

Analysis of 1,421 publications revealed significant geographical disparities in research output, with China and the United States leading contributions. Institutionally, the University of Adelaide, Zhejiang University, and Shanghai Jiao Tong University were prominent contributors. Authorship analysis identified Hannah R. Wardill as the most prolific author, while the *International Journal of Molecular Sciences* emerged as a leading journal. Rapidly growing frontiers include “proliferation,” “inhibition,” “immunotherapy,” “drug delivery,” and “tumorigenesis.”

**Discussion:**

This study provides a comprehensive overview of research trends and highlights emerging directions, aiming to advance scientific and clinical applications of gut microbiota in gastrointestinal tumor therapy.

## Introduction

Globally, cancers of the gastrointestinal (GI) tract represented around 26% of the total cancer incidence and approximately 35% of cancer-related deaths in 2018 (Siegel et al., 2022). Projections indicate that by 2040, the global burden of GI malignancies will reach 7.5 million new cases, with 5.6 million resulting in mortality (Arnold et al., 2020). The lifetime risk of developing and succumbing to gastrointestinal cancers from birth was estimated at 8.20% (95% CI 8.18–8.21) and 6.17% (6.16–6.18) in 2020. Colorectal cancer (CRC) posed the highest risk, constituting 38.5% of the lifetime risk of diagnosis and 28.2% of mortality from gastrointestinal cancers, followed by cancers of the stomach, liver, esophagus, pancreas, and gallbladder (Wang S. et al., 2024). Notably, the incidence of GI cancers is on the rise among younger adults (Ben-Aharon et al., 2023). While advancements in colorectal cancer screening have been made, the highest burden of GI cancers—such as stomach, liver, esophageal, and gallbladder cancers—was recorded in East Asia (Huang et al., 2023). The etiological factors contributing to GI tract cancers include infectious agents (e.g., Helicobacter pylori, Epstein-Barr virus), genetic predispositions (e.g., CDH1 mutations), and environmental influences (e.g., unhealthy dietary practices), which remain prevalent (Chong et al., 2024).

Cancer development is a gradual process marked by genetic and epigenetic changes, influenced significantly by the tumor microenvironment (TME), which comprises diverse cell types including immune cells and fibroblasts (Rauth et al., 2024). A unique aspect of some GI cancer TMEs is the presence of microbiota, particularly gut microbiota, which interacts complexly with the immune system (Fernandes et al., 2022). Research has revealed a complex interplay between gut microbiota and the immune system, with specific microorganisms such as *Enterococcus faecalis* and *Fusobacterium nucleatum* being linked to chronic inflammation and tumorigenesis (Liu et al., 2021). Notably, studies indicate that *F. nucleatum* can enhance chemoresistance in colorectal cancer by activating autophagy-related pathways (Yu et al., 2017). The gut microbiome also impacts other organs, as seen in gastric cancer, where H. pylori plays a significant role in disease pathogenesis (Zhao et al., 2022a). Distinct microbial communities have been identified in esophageal cancers as well (Moe and Tan, 2024). The liver, connected to the intestine via portal circulation, both influences and is influenced by gut microbiota, with dysbiosis linked to chronic inflammation and liver cancer progression (Dapito et al., 2012; Schnabl and Brenner, 2014). Beyond the microorganisms, metabolites like secondary bile acids, short-chain fatty acids, and glucuronidase contribute to a supportive TME and influence tumor development (Fu et al., 2024).

Emerging research underscores the significant role of gut microbiota in shaping therapeutic outcomes for gastrointestinal tumors, particularly in chemotherapy, immunotherapy, radiotherapy, and surgical resection. Gut microbiota can modulate the effectiveness of chemotherapeutic agents, such as 5-fluorouracil and oxaliplatin, through mechanisms like microbial translocation and immunomodulation (García-González et al., 2017; Iida et al., 2013). Notably, butyrate, a gut microbial metabolite, enhances oxaliplatin efficacy by regulating CD8 + T cell function in the tumor microenvironment (He et al., 2021). Current research is exploring the use of well-balanced microbial consortia, such as VE800, a probiotic cocktail of 11 strains, in combination with nivolumab in a Phase I/II trial for microsatellite stable colorectal cancer (MSS CRC) (Tanoue et al., 2019). Additionally, a 30-bacteria consortium (MET4) has been shown to be safe and capable of modifying gut microbiota and serum metabolome in ICB-naive patients, reinforcing the potential benefits of microbial consortia (Spreafico et al., 2023). A study also demonstrated that a mix of four Clostridiales strains (CC4) could prevent and treat colorectal cancer in mice, with the effect dependent on CD8 + T cell activation (Montalban-Arques et al., 2021). Furthermore, fecal microbiota transplantation (FMT) has shown promise in improving treatment responses when combined with PD-1 inhibitors (Huang et al., 2022b), while elevated levels of gut bacteria like *Enterococcus faecalis* may serve as indicators for early detection of anastomotic leaks (Komen et al., 2014), further highlighting the clinical relevance of gut microbiota in oncology.

Since the early 2010s, numerous studies have documented microbial dysbiosis in cancer patients, highlighting the potential role of the microbiome in cancer initiation, progression, and treatment response (LaCourse et al., 2021). While this research area is still developing, understanding the microbiota’s contributions offers substantial translational potential for improving clinical outcomes. Given the increasing complexity and breadth of microbiome research, bibliometric analysis emerges as a crucial tool for systematically assessing the evolving landscape of gut microbiota in the treatment of gastrointestinal tumors. This study will utilize bibliometric techniques to explore trends, identify key areas of focus, and delineate future research directions from 2004 to 2024, providing a knowledge map that captures the dynamic interplay between gut microbiota and cancer therapies.

## Materials and methods

### Data retrieval

**FIGURE 1:**
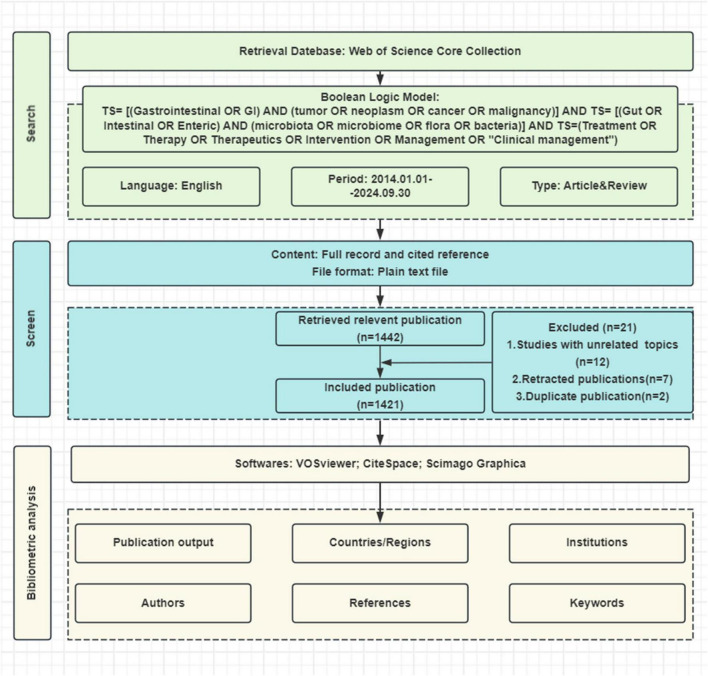
Flowchart of the literature screening process. Systematic selection process for studies on gut microbiota and gastrointestinal tumors from Web of Science (2014–2024). Final analysis included 1,051 publications after applying exclusion criteria.

Bibliometric data were collected from the Web of Science Core Collection (WoSCC) database on September 30, 2024, using the following keyword query: (TS = [(Gastrointestinal OR GI) AND (tumor OR neoplasm OR cancer OR malignancy AND TS = [(Gut OR Intestinal OR Enteric) AND (microbiota OR microbiome OR flora OR bacteria)] AND TS = (Treatment OR Therapy OR Therapeutics OR Intervention OR Management OR “Clinical management”). The search was limited to English articles and reviews published between January 1, 2014, and September 30, 2024, resulting in 1,421 relevant papers (Figure 1).

### Data processing

For the identified studies, full records and cited references were exported in both plain text and tab-delimited formats. The plain text files were analyzed using CiteSpace (version 6.1.R6), while the tab-delimited files were processed with VOSviewer (version 1.6.20.0).

### Data analysis

This research employed three bibliometric tools for a comprehensive examination of the findings. Specifically, CiteSpace facilitated co-occurrence, cluster, and emergent analyses, while VOSviewer was used for co-occurrence and cluster analyses. The OALM platform supported relational network analysis. Furthermore, the study documented journal names, impact factors (IF), and journal rankings (Q1–Q4) based on the 2023 Journal Citation Reports (JCR). Microsoft Excel was utilized to illustrate global production and trends of relevant papers, as well as to create charts reflecting various rankings.

### Research ethics

The data sources for this study were obtained from publicly available databases, making ethics committee approval unnecessary.

## Results

### Analysis of annual publications

**FIGURE 2:**
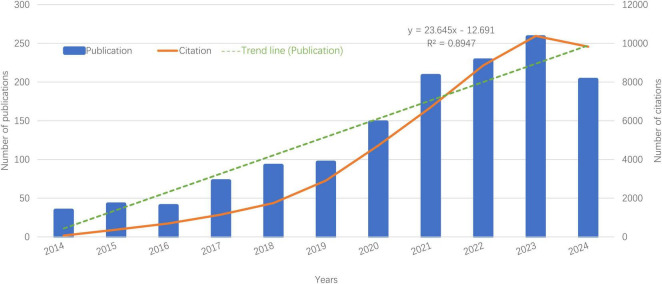
Publication and citation by year. Bar graph shows exponential growth in publications post-2020 (*R*^2^ = 0.89). Citation counts (line) correlate with rising research output.

In the past 10 years, research on the role of gut microbiota in treating gastrointestinal tumors has evolved through two distinct phases. Between 2014 and 2019, there was moderate growth, with fewer than 100 publications annually. In contrast, from 2020 to 2024, the field experienced a dramatic surge, resulting in a total of 1,051 papers—accounting for 73.96% of the decade’s research output. This growing interest is illustrated in Figure 2, which shows a polynomial curve fitting score of 0.8947, indicating a heightened global academic focus on the application of gut microbiota in gastrointestinal cancer.

### Analysis of countries/regions

Research on the role of gut microbiota in treating gastrointestinal tumors has seen contributions from a total of 92 countries and regions. China stands out as the foremost contributor, with 448 publications and a citation count of 12,905. The United States follows closely, having published 354 papers but achieving the highest citation total of 19,202. Italy ranks third, with 106 publications and 4,155 citations, as detailed in Table 1.

**TABLE 1:** The top 10 countries regions in terms of publications.

Rank	Countries	Counts	Citations	TLS	Centrality
1	China	448	12,905	128	0.08
2	United States	354	19,202	241	0.04
3	Italy	106	4,155	104	0.2
4	Australia	83	2,524	84	0.14
5	England	64	5,120	87	0.16
6	India	64	1,095	49	0
7	Canada	53	1,813	60	0.03
8	Germany	50	3,425	98	0.32
9	Japan	47	1,930	29	0.25
10	South Korea	45	1,172	17	0.04

**FIGURE 3:**
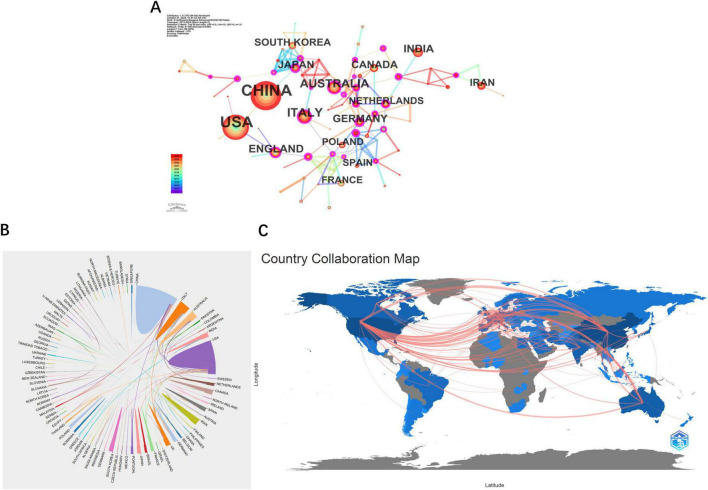
The cooperation network map of countries. **(A)** The cooperation network map of countries. **(B)** The cooperation network diagram between countries. **(C)** Geographical distribution of research output. **(A)** Node size = publication volume (China: 448; United States: 354). Purple rings = high centrality (Germany: 0.32). **(B)** Chord diagram highlights China-USA collaboration dominance.

In the visual analysis conducted using CiteSpace, circles represent different countries or regions, with their sizes reflecting the volume of publications. Lines connecting the circles indicate collaborative relationships. Nodes highlighted with purple rings signify high centrality, with the thickness of the ring indicating the level of centrality. Among the countries, Germany leads with the highest centrality score of 0.32, followed by Japan at 0.25, Italy at 0.20, the Netherlands at 0.17, and England at 0.16, as illustrated in Figure 3A. Additionally, Figures 3B,C depicts strong collaborative ties between China and the United States, while partnerships among other countries appear to be more scattered.

### Analysis of institution

A total of 2,241 institutions have contributed to the publication landscape in this field, with the 10 most active highlighted in Table 2. The University of Adelaide leads with 22 publications and 376 citations, closely followed by Zhejiang University with 21 publications and 909 citations, and Shanghai Jiao Tong University with 20 publications and the highest citation total of 2,011. The University of Adelaide also has the highest Total Link Strength (TLS), positioning it as a central node in fostering collaboration within the research community.

**TABLE 2:** The top 10 institutions in terms of publications.

Rank	Institutions	Counts	Citations	TLS	Centrality
1	University of Adelaide	22	376	38	0.06
2	Zhejiang University	21	909	16	0.04
3	Shanghai Jiao Tong University	20	2,011	16	0.04
4	Harvard Medical School	17	1,152	32	0.02
5	Chinese Academy of Sciences	17	413	22	0.06
6	Sun Yat-sen University	16	737	14	0.02
7	Sichuan University	16	359	3	0.02
8	The Chinese University of Hong Kong	15	204	9	0.03
9	University of South Australia	14	296	22	0.05
10	Nanjing Medical University	13	202	12	0.02

**FIGURE 4:**
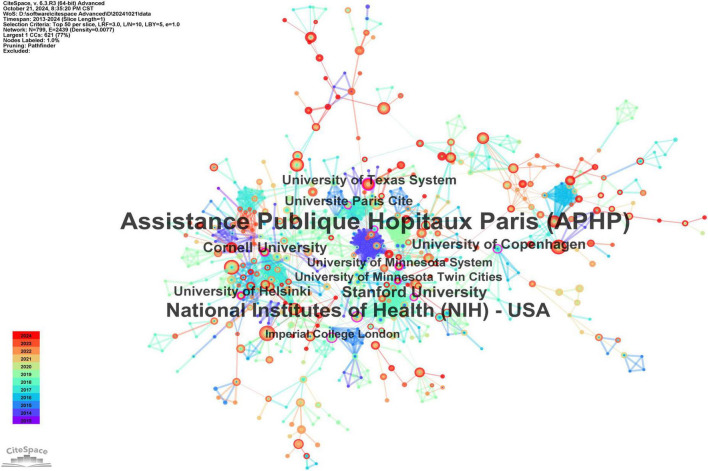
The cooperation network map of institutions. Leading institutions: University of Adelaide (22 publications), Zhejiang University (21). APHP (centrality = 0.38) and NIH (0.18) are key hubs.

The institutions exhibiting the greatest centrality, which reflects their crucial role in the collaboration network, include Assistance Publique Hôpitaux de Paris (APHP) at 0.38, National Institutes of Health (NIH) at 0.18, Stanford University at 0.14, Cornell University at 0.13, and the University of Texas System at 0.12, as illustrated in Figure 4.

### Analysis of journals

A total of 660 journals have contributed to research in this field, with 10 of them publishing more than 16 papers each, as outlined in Table 3. The *International Journal of Molecular Sciences* leads in output, followed by *Cancers* and *Nutrients*. Among the top 10 journals, *Frontiers in Immunology* has the highest Impact Factor (IF) at 5.7, ranking fifth in publication volume. All these prolific journals are classified as Q1 and Q2 according to the 2023 Journal Citation Reports (JCR), reflecting their significant academic impact.

**TABLE 3:** The top 10 journals and co-cited journals in terms of publications.

Rank	Journals	Count	IF	JCR	Rank	Co-cited journals	Citations	IF	JCR
1	International Journal of Molecular Sciences	44	4.9	Q2	1	Nature Reviews Gastroenterology & Hepatology	2,776	45.9	Q1
2	Cancers	40	4.5	Q1	2	Gut	1,625	23.0	Q1
3	Nutrients	39	4.8	Q1	3	Nutrients	1,426	4.8	Q1
4	Frontiers in Microbiology	28	4.0	Q1	4	International Journal of Molecular Sciences	1,117	4.9	Q2
5	Frontiers in Immunology	25	5.7	Q1	5	World Journal of Gastroenterology	1,025	4.3	Q2
6	Frontiers in Pharmacology	21	4.4	Q1	6	Alimentary Pharmacology & Therapeutics	895	6.6	Q1
7	Frontiers in Cellular and Infection Microbiology	19	4.6	Q2	7	Frontiers in Microbiology	883	4.0	Q1
8	Frontiers in Oncology	18	3.5	Q2	8	Cancers	706	4.5	Q1
9	Microorganisms	17	4.1	Q2	9	Frontiers in Pharmacology	536	4.4	Q1
10	World Journal of Gastroenterology	16	4.3	Q2	10	Scientific Reports	534	3.8	Q2

Co-citation relationships arise when journals are cited together in the same work, indicating a link in the quality of scholarly content. As shown in Table 3, the top three co-cited journals are *Nature Reviews Gastroenterology & Hepatology*, *Gut*, and *Nutrients*, with all top 10 co-cited journals also categorized as Q1 and Q2.

### Analysis of authors and co-cited authors

Analyzing authors is essential for identifying key contributors within a research field. Table 4 presents the top 10 authors in the study of gut microbiota’s role in treating gastrointestinal tumors, showcasing their impressive productivity and citation rates. Notably, Hannah R. Wardill from the University of South Australia leads in publications with 14 papers, while Antonio Gasbarrini from Università Cattolica del Sacro Cuore in Rome stands out with the highest citation count of 697.

**TABLE 4:** The top 10 authors and co-cited authors in terms of publications.

Rank	Author	Count	H-index	Rank	Co-cited author	Citations	H-index
1	Wardill, Hannah R.	14	24	1	Gasbarrini, Antonio	697	98
2	Gasbarrini, Antonio	12	98	2	Stadlbauer, Vanessa	649	42
3	Bowen, Joanne M.	10	46	3	Sfanos, Karen S.	363	29
4	Kazmierczak-Siedlecka, Karolina	8	18	4	Bowen, Joanne M.	215	46
5	Ianiro, Gianluca	7	50	5	Wardill, Hannah R.	213	24
6	Yu, Jun	6	107	6	li, qi	178	3
7	Skonieczna-Zydecka, Karolina	6	28	7	Ianiro, Gianluca	168	50
8	Sfanos, Karen S.	5	29	8	Kazmierczak-Siedlecka, Karolina	139	18
9	Stadlbauer, Vanessa	5	42	9	Chen, Wei	134	39
10	Baba, Hideo	5	84	10	Cammarota, Giovanni	110	10

**FIGURE 5:**
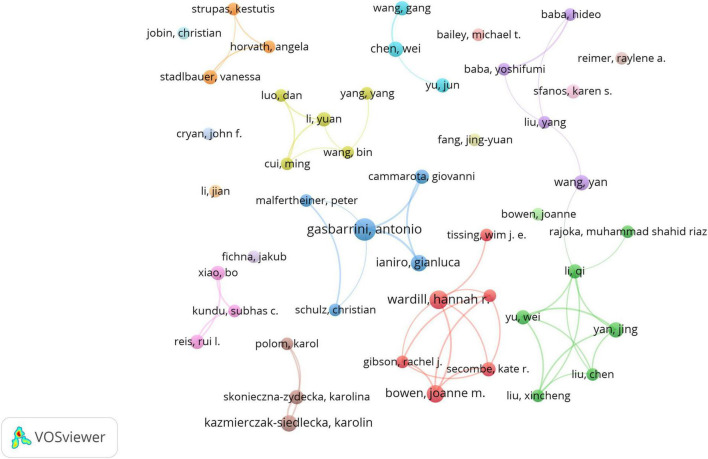
The cooperation network map of authors. Hannah R. Wardill (14 papers) and Antonio Gasbarrini (697 citations) are top contributors. Dispersed clusters reflect limited core author cohesion.

Using VOSviewer, we identified 8,364 researchers contributing to this area. Following Price’s law, which categorizes authors with more than three publications as core authors, we identified 146 core authors, as illustrated in Figure 5. However, these core authors collectively published only 527 papers, representing 37.09% of the total sample. This figure is significantly below 50%, indicating that scholars in this field are widely dispersed and have yet to form a cohesive core author group. This lack of collaboration may have somewhat impeded academic progress regarding the role of gut microbiota in treating gastrointestinal tumors.

### Analysis of co-cited references and references bursts

Among the 724 co-cited references, we identified the top 10, detailed in Table 5. The work by Routy et al. (2018), titled “Gut microbiome influences efficacy of PD-1-based immunotherapy against epithelial tumors,” published in *Science*, stands out with the highest citation count of 162. This study demonstrated that primary resistance to immune checkpoint inhibitors (ICIs) can be linked to an abnormal gut microbiome composition. It was found that antibiotics diminished the clinical benefits of ICIs in patients with advanced cancer. Additionally, FMT from cancer patients who responded to ICIs into germ-free or antibiotic-treated mice enhanced the antitumor effects of PD-1 blockade, whereas FMT from non-responding patients did not yield similar results.

**TABLE 5:** The top 10 co-cited references in terms of publications.

Rank	Co-cited references	Citations	IF	JCR	Centrality
1	Routy B, Le Chatelier E, Derosa L, et al. Gut microbiome influences efficacy of PD-1-based immunotherapy against epithelial tumors. Science. 2018;359(6371):91-97.	162	44.7	Q1	0.08
2	Gopalakrishnan V, Spencer CN, Nezi L, et al. Gut microbiome modulates response to anti-PD-1 immunotherapy in melanoma patients. Science. 2018;359(6371):97-103.	135	44.7	Q1	0.06
3	Matson V, Fessler J, Bao R, et al. The commensal microbiome is associated with anti-PD-1 efficacy in metastatic melanoma patients. Science. 2018;359(6371):104-108.	109	44.7	Q1	0.11
4	Alexander JL, Wilson ID, Teare J, Marchesi JR, Nicholson JK, Kinross JM. Gut microbiota modulation of chemotherapy efficacy and toxicity. Nat Rev Gastroenterol Hepatol. 2017;14(6):356-365.	55	45.9	Q1	0.01
5	Sung H, Ferlay J, Siegel RL, et al. Global Cancer Statistics 2020: GLOBOCAN Estimates of Incidence and Mortality Worldwide for 36 Cancers in 185 Countries. CA Cancer J Clin. 2021;71(3):209-249.	50	503.1	Q1	0
6	Tanoue T, Morita S, Plichta DR, et al. A defined commensal consortium elicits CD8 T cells and anti-cancer immunity. Nature. 2019;565(7741):600-605.	48	50.5	Q1	0.07
7	Pushalkar S, Hundeyin M, Daley D, et al. The Pancreatic Cancer Microbiome Promotes Oncogenesis by Induction of Innate and Adaptive Immune Suppression [published correction appears in Cancer Discov. 2020 Dec;10(12):1988.	48	29.7	Q1	0.22
8	Sivan A, Corrales L, Hubert N, et al. Commensal Bifidobacterium promotes antitumor immunity and facilitates anti-PD-L1 efficacy. Science. 2015;350(6264):1084-1089.	48	44.7	Q1	0.07
9	Geller LT, Barzily-Rokni M, Danino T, et al. Potential role of intratumor bacteria in mediating tumor resistance to the chemotherapeutic drug gemcitabine. Science. 2017;357(6356):1156-1160.	47	44.7	Q1	0.17
10	Helmink BA, Khan MAW, Hermann A, Gopalakrishnan V, Wargo JA. The microbiome, cancer, and cancer therapy. Nat Med. 2019;25(3):377-388.	47	58.7	Q1	0.12

**FIGURE 6:**
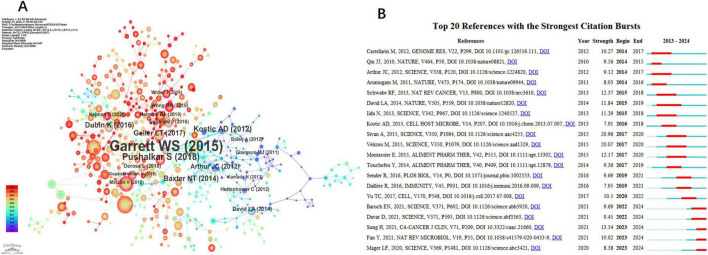
Major References and Top 20 References with Strong Citation Bursts. **(A)** Co-citation network clusters: microbiome-driven carcinogenesis (Garrett et al., 2015). **(B)** Top citation bursts: Sivan et al. (2015; strength = 20.96). **(A)** The Co-cited References network. **(B)** Top 20 references with the strongest citation bursts.

Figure 6A displays the network visualization map of co-cited references, with a Q-value of 0.8658 and a mean S value of 0.947. The top three references based on centrality are led by Garrett et al. (2015), with a centrality score of 0.35, followed by Pushalkar et al. (2018) with a score of 0.22, and Kostic et al. (2012) with a score of 0.19. Garrett et al.’s paper explores how microbes and the microbiota can influence carcinogenesis, the effectiveness of cancer therapies, and cancer-related complications. Pushalkar S et al. discovered that the cancerous pancreas has a significantly more abundant microbiome compared to the normal pancreas in both mice and humans, with certain bacteria being notably increased in the tumorous pancreas relative to the gut. They also found that microbiome ablation protects against preinvasive and invasive pancreatic ductal adenocarcinoma (PDA) and enhances the efficacy of checkpoint-targeted immunotherapy by upregulating PD-1 expression. Lastly, Kostic et al.’s research presents genomic analysis revealing a significant enrichment of Fusobacterium species in colorectal carcinomas, particularly phylotypes closely related to *F. nucleatum*, *F. mortiferum*, and *F. necrophorum*. Their analysis also indicates broader alterations in the tumor environment, such as the depletion of the *Bacteroidetes* and *Firmicutes phyla*, notably the order Clostridiales.

A paper exhibiting a strong citation burst marks a significant milestone in the field, reflecting widespread recognition and impact. Figure 6B presents the top 20 references with the highest citation bursts, highlighting the time interval from 2013 to 2024 in blue, while the burst duration is shown in red. The article “Commensal *Bifidobacterium* promotes antitumor immunity and facilitates anti-PD-L1 efficacy” by Sivan et al. (2015) exhibited the strongest citation burst (strength 20.96). Furthermore, ongoing citation bursts are evident in specific articles, including works by Erez N. Baruch et al., Diwakar Davar et al., Hyuna Sung et al., Yong Fan et al., and Lukas F. Mager et al. This suggests that these research topics are likely to maintain their prominence in the future and may emerge as potential frontiers in studying the role of gut microbiota in treating gastrointestinal tumors.

### Analysis of keywords

Keywords serve as concise summaries, highlighting the main themes of a document and offering a representative overview of its scientific content. Analyzing these keywords can uncover focal areas within a research field. Table 6 lists the top 20 most frequently occurring terms in this domain, with “gut microbiota” emerging as the most prevalent keyword.

**TABLE 6:** The top 20 keywords in terms of publications.

Rank	Keywords	Count	Centrality	Rank	Keywords	Count	Centrality
1	Gut microbiota	569	0.07	11	Bacteria	106	0.14
2	Colorectal cancer	301	0	12	Cell	103	0.07
3	Intestinal microbiota	187	0.03	13	Double blind	96	0.04
4	Inflammatory bowel disease	181	0.04	14	*Fusobacterium nucleatum*	95	0.06
5	Cancer	138	0.03	15	Probiotics	88	0.01
6	Inflammation	137	0.01	16	Fecal microbiota	86	0
7	Gut microbiome	132	0.04	17	Therapy	83	0
8	Ulcerative colitis	123	0.22	18	Microbiota	82	0.05
9	Chain fatty acids	117	0	19	Expression	76	0.04
10	Risk	114	0.03	20	Gastrointestinal tract	72	0.18

**FIGURE 7:**
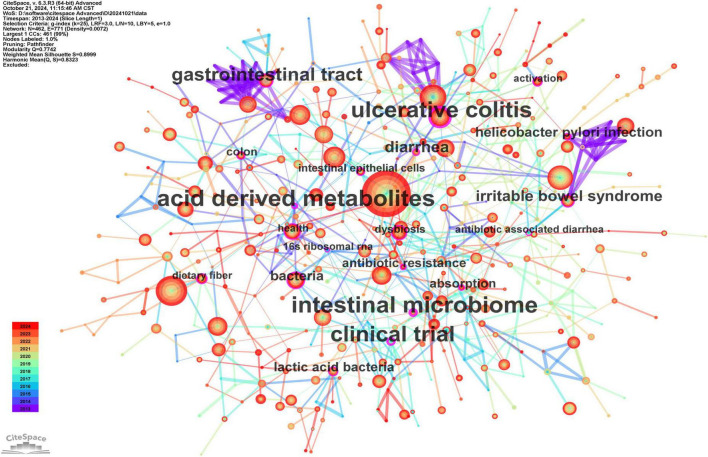
The main keywords. “Gut microbiota” (682 mentions) links to “immunotherapy” and “dysbiosis.” High centrality: “acid-derived metabolites” (0.23).

The co-occurring keyword analysis conducted in CiteSpace, covering the period from 2013 to 2024 at yearly intervals, reveals a network visualized in Figure 7. This network consists of 462 nodes and 771 links, illustrating strong correlations among keywords. Node size reflects frequency, while line color indicates chronology, transitioning from blue (older) to orange (newer). Notably, the top three keywords in terms of centrality, represented by the thickness of the purple rings, are “acid-derived metabolites” (centrality: 0.23), “intestinal microbiome” (centrality: 0.23), and “ulcerative colitis” (centrality: 0.22). These high-centrality nodes highlight their significant influence, representing emerging trends in the research on gut microbiota’s role in treating gastrointestinal tumors.

**FIGURE 8:**
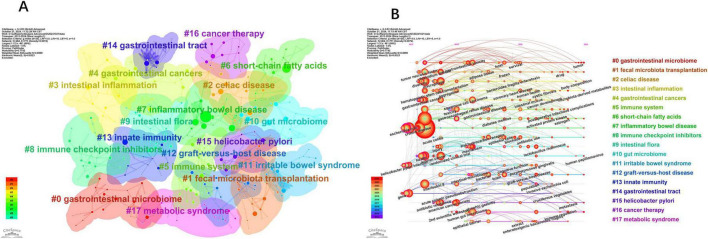
The main keywords clusters. **(A)** 17 thematic groups (Q = 0.77), e.g., “fecal microbiota transplantation.” **(B)** Timeline shows sustained focus on metabolites and immunotherapy. **(A)** Keywords cluster analysis co-occurrence map. **(B)** Timeline of keywords cluster.

Clustering analysis was performed on co-occurring keywords, resulting in 17 clusters with a quality index (Q) of 0.7742 and a silhouette score (S) of 0.8999, indicating reliable and meaningful clustering outcomes (Figure 8A). The cluster labels reveal the major themes within the research field. Figure 8B, created by sorting Figure 8A by time period, illustrates the historical development of research on gut microbiota’s role in treating gastrointestinal tumors. The core terms of each cluster exhibit varying levels of interest over time. Some topics have persisted and evolved, leading to new research directions, such as gastrointestinal microbiome (#0), fecal microbiota transplantation (#1), celiac disease (#2), intestinal inflammation (#3), short-chain fatty acids (#6), inflammatory bowel disease (#7), immune checkpoint inhibitors (#8), gut microbiome (#10), graft-versus-host disease (#12), Helicobacter pylori (#15), and cancer therapy (#16). In contrast, other topics have gradually lost prominence, including gastrointestinal cancers (#4), immune system (#5), intestinal flora (#9), irritable bowel syndrome (#11), innate immunity (#13), gastrointestinal tract (#14), and metabolic syndrome (#17).

**FIGURE 9:**
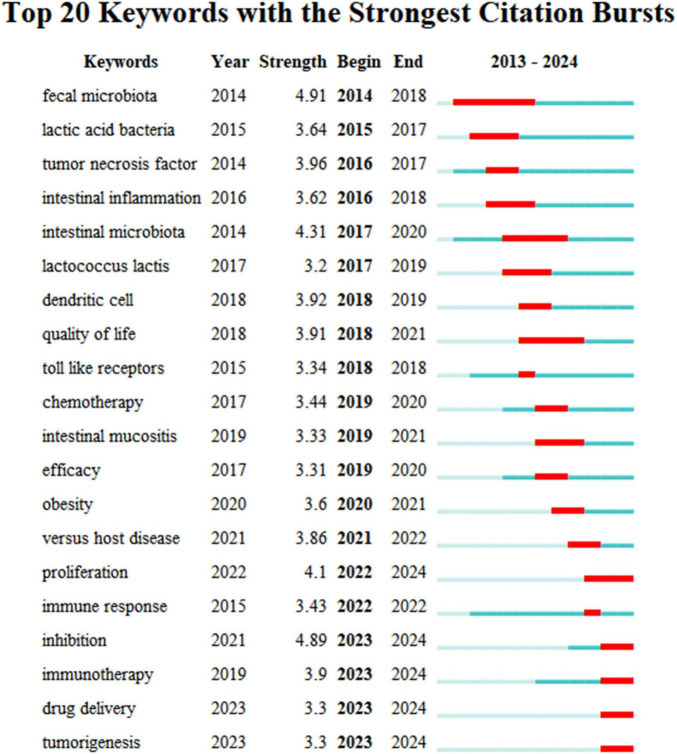
Top 20 Keywords with the Strongest Citation Bursts. Strongest burst: “fecal microbiota” (4.91). Emerging trends: “immunotherapy” and “tumorigenesis” (2022–2024).

Using the keyword co-citation network, we conducted an emergent word detection analysis and present the top 20 keywords with the strongest citation bursts in this field in Figure 9. The keyword “fecal microbiota” recorded the most significant citation burst, with a score of 4.91. Additionally, keywords such as “proliferation,” “inhibition,” “immunotherapy,” “drug delivery,” and “tumorigenesis” have shown continued burstiness through 2024, indicating that these research directions are likely to maintain momentum in the future.

## Discussion

### General information

Interest in the role of gut microbiota in the treatment of gastrointestinal tumors has surged over the past decade, as evidenced by increasing annual publication rates and citation numbers. Since 2019, the field has experienced significant clinical progress, including fecal metagenomic and metabolomic studies on samples. These investigations have revealed two distinct patterns of microbiome elevation, featuring species such as *Fusobacterium nucleatum* and *Actinomyces odontolyticus*, as well as increased levels of branched-chain amino acids, phenylalanine, and bile acids, including deoxycholate. These changes can occur early in the development of colorectal cancer, suggesting potential etiological and diagnostic relevance (Yachida et al., 2019). Sequencing studies have uncovered alterations in microbial composition and ecology in patients with CRC, while functional studies using animal models have identified specific bacteria, such as *Fusobacterium nucleatum* and certain strains of *Escherichia coli* and *Bacteroides fragilis*, as playing pivotal roles in colorectal carcinogenesis (Wong and Yu, 2019). Additionally, a population-based study in a high-risk area for gastric cancer highlighted Helicobacter pylori as a key contributor to gastric microbial dysbiosis. Remarkably, successful eradication of H. pylori has been shown to restore gastric microbiota to levels comparable to those of negative controls and provides greater benefits to gut microbiota than unsuccessful treatments, including an increase in probiotics and a potential reduction in drug-resistance mechanisms (Guo Y. et al., 2020).

The results indicate that China leads with 448 research publications from 2014 to 2024, followed closely by the United States with 354 publications. Both nations dominate the list of the top 10 most productive institutions. This leadership can be attributed to specific situational requirements and significant investments. In China, 41.6% of new cancer cases and 49.3% of cancer-related deaths are associated with cancers of the digestive system (He et al., 2024). While the incidence and mortality rates of CRC have declined in certain European and North American countries, they continue to rise in China (Li et al., 2021). Notably, early-onset CRC is also increasing in the United States, despite substantial declines in older age groups (Siegel et al., 2019).

Among the top 10 journals with the highest publication counts, the *International Journal of Molecular Sciences* takes the lead, serving as an international, open-access platform primarily focused on research in molecular sciences. Co-citation analysis predominantly features high-impact factor (IF) and Q1 journals, with *Nature Reviews Gastroenterology & Hepatology* achieving an impressive IF of 45.9. The presence of numerous high-quality, impactful journals underscores the significant interest in research regarding the role of gut microbiota in treating gastrointestinal tumors. This information will aid future scholars in selecting suitable journals for manuscript submissions related to this field.

Hannah R. Wardill from the University of South Australia leads the publication count with 14 papers, focusing on topics such as mucositis. Their research updates the understanding of the pathogenesis of mucositis and reviews management strategies during chemotherapy (Bowen et al., 2019; Van Sebille et al., 2015), as well as investigates the protective mechanisms of probiotics in reducing radiation-induced oral mucositis (Wardill et al., 2024). In contrast, Antonio Gasbarrini from Università Cattolica del Sacro Cuore in Rome stands out with the highest citation count of 697. Contributions include important works such as “Management of Helicobacter pylori Infection: The Maastricht V/Florence Consensus Report” and the “European Consensus Conference on Fecal Microbiota Transplantation in Clinical Practice.” (Cammarota et al., 2017; Malfertheiner et al., 2017) Gasbarrini’s research emphasizes the mutualistic relationship between variations in gut microbiota and various diseases,(Rinninella et al., 2019) as well as the role of commensal Clostridia as key players in maintaining gut homeostasis (Lopetuso et al., 2013).

### Research hotspots

Through reference co-citation analysis, the key research directions and developments in this field have been clarified. The top three most cited articles, all published in *Science*, demonstrate that gut microbiota composition significantly influences the efficacy of immune checkpoint inhibitors. These studies shows that FMT from cancer patients who responded to ICIs enhances the effects of PD-1 blockade in germ-free or antibiotic-treated mice, while FMT from non-responders does not (Routy et al., 2018). Although PD-1 inhibitors are used across various cancers, response rates remain low, prompting investigations into combinations of PD-1/PD-L1 inhibitors with other treatments to improve outcomes (Huang et al., 2022a). The role of gut microbiota in cancer immunotherapy is gaining attention. Species like *Bacteroides fragilis*, *Bifidobacterium*, *Akkermansia muciniphila*, and *Faecalibacterium* spp. have shown positive effects in both preclinical and clinical studies (Gopalakrishnan et al., 2018; Sivan et al., 2015; Vétizou et al., 2015). Combining FMT with anti-PD-1 therapy appears safe in melanoma treatment, as it can alter the gut microbiome and potentially reprogram the tumor environment to overcome resistance (Cancer Discover, 2023; Davar et al., 2021). MSS CRCs often resist anti-PD-1 therapy. FMT from *Fusobacterium nucleatum*-high MSS CRC patients to germ-free mice has been shown to increase sensitivity to PD-1 inhibitors (Wang X. et al., 2024). A clinical trial combining an anti-PD-1 inhibitor with FMT from responders involved 13 patients with advanced solid tumors resistant to anti-PD-1 treatment. This strategy resulted in notable microbiota changes and clinical benefits for six patients, including one partial response and five stable diseases, leading to an objective response rate of 7.7% and a disease control rate of 46.2%. These findings suggest that FMT with beneficial microbiota may help address resistance to anti-PD-1 inhibitors, particularly in advanced gastrointestinal cancers (Kim et al., 2024).

While the identified hotspots—such as the interplay between specific microbiota (e.g., *Fusobacterium nucleatum*, *Escherichia coli*) and therapeutic outcomes—highlight promising translational avenues, they also underscore critical challenges in the field. For instance, the dual role of pathobionts like *F. nucleatum*, which can both drive tumorigenesis (Kostic et al., 2013) and enhance immunotherapy efficacy (Gao et al., 2021), exemplifies the complexity of microbiota-tumor interactions. This duality necessitates a nuanced understanding of microbial ecology within the TME and its spatiotemporal dynamics (Nejman et al., 2020). The clinical success of FMT and probiotics in overcoming chemoresistance or ICB resistance, as demonstrated in recent trials [e.g., improved survival in MSS-CRC with FMT combinations (Zhao et al., 2023)], signals a paradigm shift toward microbiome-targeted adjuvant therapies. However, the variability in donor microbiota efficacy and safety risks, such as pathogen transmission (DeFilipp et al., 2019), emphasize the need for standardized protocols and personalized microbiota profiling (Mirzayi et al., 2021).

Furthermore, the emphasis on microbial metabolites (e.g., SCFAs, inosine) as immunomodulators reveals a broader mechanistic convergence between microbiome research and cancer metabolism. These metabolites not only enhance cytotoxic T cell function (He et al., 2021) but also reprogram immunosuppressive myeloid cells (Lam et al., 2021), offering combinatorial strategies to reshape the TME. Yet, the translation of preclinical findings [e.g., butyrate’s context-dependent pro- or anti-tumor effects (Yang et al., 2021)] into clinical practice remains hindered by interpatient microbial heterogeneity (Allaband et al., 2024) and the lack of biomarkers to predict therapeutic responses (Schupack et al., 2022).

Centrality serves as an important metric for evaluating the significance and influence of nodes within a research domain. By analyzing keyword centrality, we can also identify potential research hotspots. In this study, the top three keywords with high centrality are “acid-derived metabolites,” “intestinal microbiome,” and “ulcerative colitis.”

Acid-derived metabolites are a diverse group of organic compounds produced through metabolic processes involving acids. They can originate from the breakdown of various substances, including amino acids, fatty acids, and carbohydrates. Certain gut bacteria, such as *Faecalibacterium*, *Roseburia*, *Bifidobacterium*, *Eubacterium*, and *Lactobacillus*, can ferment dietary fibers to produce short-chain fatty acids (SCFAs) that have protective effects on the gut and are negatively associated with CRC (Coker et al., 2022). The SCFA butyrate enhances immune checkpoint blockade (ICB) responses by inhibiting histone deacetylase activity in CD8 + T cells and inducing the expression of inhibitor of DNA binding 2 (ID2), which boosts T cell activation and mitigates T cell exhaustion (He et al., 2021). Additionally, butyrate and pentanoate improve the effector functions of CAR T cells *in vitro* and increase their activity in mouse models (Luu et al., 2021). Furthermore, butyrate has been shown to enhance radiotherapy-induced abscopal effects in mice by promoting cross-presentation by dendritic cells, thereby facilitating CD8 + T cell-mediated clearance of tumors that are not directly irradiated (Uribe-Herranz et al., 2020). However, another study suggested that butyrate might reduce radiotherapy efficacy by suppressing type I interferon responses in dendritic cells (Yang et al., 2021). SCFAs can also activate regulatory T (Treg) cells, which may suppress antitumor immune responses in later stages of tumorigenesis (Smith et al., 2013).

Metabolites derived from dietary tryptophan produced by the microbiota also exhibit potent immunomodulatory effects (Blake et al., 2024). For example, oral gavage of mice with *Lactobacillus* reuteri (but not *L. johnsonii*) effectively controlled the growth of B16F10 melanoma tumors and enhanced anti-PD-L1 therapy (Bender et al., 2023). Conversely, tryptophan metabolites from indole-producing bacteria, including *Lactobacillus* spp., have been found to suppress ICB responses in mice with pancreatic ductal adenocarcinoma (PDAC) through the induction of AHR signaling in macrophages (Hezaveh et al., 2022). Notably, indole-3-acetic acid (3-IAA), a metabolite derived from tryptophan and produced by Bacteroides spp., has been demonstrated to enhance the effectiveness of combination chemotherapy in pancreatic cancer (Tintelnot et al., 2023). In an orthotopic pancreatic cancer model, 3-IAA was found to increase the infiltration of neutrophils into tumors, promoting their degranulation, reactive oxygen species release, and subsequent apoptosis following chemotherapy. This process contributed to the suppression of tumor cell proliferation (Tintelnot et al., 2023).

Both SCFAs and tryptophan metabolites can help reduce the immunotoxicity associated with cancer therapies. Specifically, butyrate and propionate are critical in regulating Treg cell frequency and function in the colon, which may affect the onset and severity of colitis induced by ICB (Furusawa et al., 2013; Simpson et al., 2022). In mouse studies, a probiotic blend containing four *Bifidobacterium* species was found to lower systemic inflammatory cytokine levels and alleviate colitis caused by anti-CTLA4 treatment and dextran sodium sulfate (DSS).(Wang et al., 2018). Moreover, administration of the tryptophan metabolite indole-3-carboxaldehyde has been shown to mitigate DSS-induced colitis in mice through an AHR–IL-22 signaling pathway in the gut (Renga et al., 2022). Additionally, microbiota-mediated protection against severe hematological and gastrointestinal toxicities from radiotherapy has been linked to the SCFA propionate and tryptophan metabolites such as 1H-indole-3-carboxaldehyde and kynurenic acid (Guo H. et al., 2020). Indole-3-propionic acid has also been identified as a key factor contributing to the protective effects of FMT against toxicities induced by radiotherapy (Xiao et al., 2020).

The centrality of “acid-derived metabolites” underscores their dual role as both therapeutic enhancers and potential obstacles in cancer treatment. While preclinical studies highlight the immunomodulatory potential of SCFAs like butyrate in boosting ICB responses (He et al., 2021), their context-dependent effects—such as suppressing radiotherapy efficacy via interferon inhibition (Yang et al., 2021)—reveal a critical need for patient-specific metabolic profiling. The variability in microbial metabolite production across individuals complicates standardized therapeutic applications (Allaband et al., 2024), emphasizing the necessity for personalized microbiota modulation strategies. Furthermore, the interplay between SCFAs and Treg activation raises questions about timing and dosing in clinical interventions (Smith et al., 2013), as early-stage immune activation may conflict with late-stage immunosuppression. Future research should prioritize integrating metabolomic data with tumor genomic and immune profiles to identify predictive biomarkers (e.g., butyrate receptor expression (He et al., 2021)), enabling precision targeting of acid-derived metabolites in combination therapies.

A previous study utilized 16S rRNA profiling from the Predicting Response to Standardized Colitis Therapy (PROTECT) cohort to demonstrate that the proliferation of oral cavity-associated bacteria and a reduction in Clostridiales are associated with the progression of ulcerative colitis (UC) (Schirmer et al., 2018). This study also highlighted the increased variability in the gut microbiome over time as a factor influencing treatment effectiveness. In metagenomic studies of inflammatory bowel disease (IBD), such as the PRISM cohort, UC patients exhibited greater microbial diversity, with notable enrichments of *Bifidobacterium breve* and *Clostridium symbiosum* (Franzosa et al., 2019). Connections between microbes derived from UC patients and host immune responses include the induction of Th1 cells and gut inflammation driven by oral Klebsiella strains, as well as the activation of Th17 cells through adhesion to epithelial cells (Atarashi et al., 2015; Atarashi et al., 2017). Research has shown that nitrate reductase, which facilitates inflammation-related colonization by *Veillonella parvula*, is also present in *E. coli* and *K. pneumoniae* (Winter et al., 2013). Notably, bacteriophage depletion was observed early in UC and was correlated with disease severity. A recent study indicated that phage therapy could suppress *K. pneumoniae*, which is associated with human IBD, and reduce inflammation and disease severity in colitis-prone mice (Federici et al., 2022).

Establishing a causal relationship between gut dysbiosis and UC is highly challenging; however, this difficulty arises because UC itself is marked by significant dysbiosis, likely driven by inflammatory changes and substantial alterations in mucosal integrity characteristic of the disease process (Walters et al., 2014). Consequently, the changes in microbial composition may also stem from mucosal damage and chronic inflammation (Lupp et al., 2007). Nevertheless, direct modulation of the gut microbiota through FMT or probiotic supplementation has shown promise in managing these patients (Costello et al., 2017; Moayyedi et al., 2015; Rossen et al., 2015).

Gut dysbiosis is intricately linked to inflammation in the GI tract and plays a crucial role in the development of colitis-associated CRC (Wong and Yu, 2023). Previous meta-analyses indicate that patients with ulcerative colitis face a cumulative CRC risk of 18.4% over 30 years (Eaden et al., 2001). Research involving the transplantation of stool from CRC patients into germ-free mice has demonstrated that this can trigger colon inflammation accompanied by increased levels of pro-inflammatory cytokines (Li et al., 2019). Specific cancer-promoting pathobionts, such as *Fusobacterium nucleatum* (Kostic et al., 2013), *Peptostreptococcus anaerobius* (Long et al., 2019), enterotoxigenic *Bacteroides fragilis* (Wu et al., 2009), *Parvimonas micra* (Zhao et al., 2022b), and pks + *Escherichia coli* (Arthur et al., 2012) are linked to colonic inflammation. For certain microorganisms, like enterotoxigenic *Bacteroides fragilis*, inflammation serves as the primary mechanism driving colorectal tumorigenesis (Cao et al., 2021). In contrast, the pro-tumorigenic effects of pks + *E. coli* appear to be independent of its inflammatory potential, although inflammation may still contribute by sustaining the expression of pks-associated genes (Arthur et al., 2014).

The high centrality of “ulcerative colitis” reflects its role as a critical precursor to colitis-associated CRC, yet translating microbiome findings into clinical practice remains fraught with challenges. While dysbiosis in UC is strongly linked to CRC risk (Wong and Yu, 2023), establishing causality is hindered by the bidirectional relationship between chronic inflammation and microbial shifts (Lupp et al., 2007). Interventions like FMT show promise in restoring microbial balance and reducing inflammation (Moayyedi et al., 2015), but donor-recipient compatibility and long-term safety (e.g., pathogen transmission risks; DeFilipp et al., 2019) demand rigorous standardization. Additionally, the enrichment of pathobionts such as *Fusobacterium nucleatum* in UC-CRC progression highlights the need for targeted antimicrobial strategies (Kostic et al., 2013), though these must balance efficacy with microbiome preservation. Emerging tools like phage therapy (Federici et al., 2022) and microbial consortia engineering (Montalban-Arques et al., 2021) offer novel approaches, but their clinical scalability and ecological impact require further validation. Future studies should focus on longitudinal cohorts to disentangle cause-effect dynamics and develop microbiome-based stratification models for UC patients at high CRC risk (Chen et al., 2021), ultimately bridging the gap between mechanistic insights and therapeutic innovation.

### Future trends

Burst detection analysis reveals emerging research trends by pinpointing keywords that experience significant citation spikes, reflecting periods of heightened academic interest. Recent trends in the role of gut microbiota in treating gastrointestinal tumors have been identified based on the latest keyword bursts, including “proliferation,” “inhibition,” “immunotherapy,” “drug delivery,” and “tumorigenesis.”

### “Proliferation” “inhibition” and “tumorigenesis”

The keywords “proliferation,” “inhibition,” and “tumorigenesis,” identified through burst detection analysis, highlight recent emerging trends in understanding the role of gut microbiota in the treatment of gastrointestinal tumors, particularly regarding their mechanisms of action on systemic immunity and cancer treatment response. This sets the stage for an exploration of how microbiota influence these processes and their effects on therapeutic outcomes.

A highly immunosuppressive TME presents a major obstacle to the effectiveness of immunotherapy. Recent research has significantly enhanced our understanding of how gut microbiota can alter the TME and influence immune responses to cancer treatments. Various mechanisms can shift the balance between a suppressive and inflammatory TME, ultimately affecting the effectiveness of cancer treatments (Blake et al., 2024). A pivotal study conducted in 2020 analyzed bacterial compositions across seven cancer types, demonstrating that these microorganisms can play conflicting roles within tumors (Nejman et al., 2020). This finding underscores the notion that intratumoral bacteria may either promote or impede cancer progression.

**A:** Microorganism-associated molecular patterns (MAMPs), including lipopolysaccharides, peptidoglycans, and flagellin, are secreted by gut bacteria and recognized by pattern recognition receptors in the innate immune system, such as Toll-like receptors (TLRs) and NOD-like receptors. These interactions can modulate immune responses both systemically and within the TME. For instance, microbiota-derived agonists of the stimulator of interferon genes (STING), such as cyclic di-AMP, enhance ICB responses by promoting type I interferon production in the TME (Lam et al., 2021). This type of interferon fosters a more immunostimulatory myeloid compartment and encourages communication between natural killer cells and dendritic cells. A high-fiber diet or supplementation with *Akkermansia muciniphila* can boost cyclic di-AMP secretion from microbiota, leading to similar TME changes that improve ICB responses (Lam et al., 2021). Additionally, *Enterococcus* spp. have been shown to enhance responses to chemotherapy and ICB in mouse melanoma models by secreting SagA, which hydrolyzes bacterial peptidoglycans to create muropeptides that activate NOD2 signaling in macrophages, triggering immune activation (Griffin et al., 2021).

**B:** Live bacteria from the gut microbiota can migrate to the TME or other organs. Many cancer therapies can increase gut barrier permeability, often referred to as “leaky gut,” which allows for the translocation of live bacteria and immunomodulatory products (Montassier et al., 2015). While this translocation can lead to infections during chemotherapy and hematopoietic cell transplantation (HCT), it may also enhance responses to chemotherapy and ICB (Peled et al., 2020). In mice, cyclophosphamide treatment was found to increase gut barrier permeability, facilitating the movement of bacteria like *Enterococcus hirae* and *Lactobacillus johnsonii* to tumor-draining lymph nodes, where they skewed CD4 + T cells in the TME toward a T helper 17 (TH17) phenotype, thereby improving tumor control (Viaud et al., 2013). Conversely, translocation of *Fusobacterium nucleatum*, typically found in the oral microbiota, to colorectal cancer tumors is associated with poor ICB responses (Jiang et al., 2023).

**C:** Immunomodulatory metabolites secreted by gut microbiota can bolster immune responses to cancer treatments by enhancing T cell function and antigen presentation. For example, *Bifidobacterium pseudolongum* boosts ICB and other immunotherapy responses by producing inosine, which activates T cell responses in the TME via adenosine A2A receptor (A2AR) signaling (Mager et al., 2020). SCFAs generated by anaerobic bacteria also significantly modulate the activities of various immune cell populations (He et al., 2021).

**D:** Immune responses can be induced against microbial antigens that cross-react with tumor-associated antigens. For instance, a study identified a prophage in the genome of *Enterococcus hirae* that encodes an major histocompatibility complex (MHC) class I-binding epitope. CD8 + T cells that are cross-reactive with this epitope demonstrated robust activity against a protein encoded by the proto-oncogene PSMB4, which markedly improved ICB-mediated tumor control in both mice and humans (Fluckiger et al., 2020). Another recent study found T cells in the TME and peripheral blood that reacted to the glioblastoma neoantigen SIN3A* as well as to peptides produced by the microbiota (Naghavian et al., 2023). The tumor microbiota may also provide non-self-antigens that T cells can recognize, thus modulating therapy responses. An analysis of metastases from 17 melanoma patients uncovered a variety of MHC class I- and II-binding peptides derived from 41 bacterial types (Kalaora et al., 2021).

**E:** Certain bacteria can promote carcinogenesis by impacting the genomic stability of host cells. Some strains produce small-molecule genotoxins that cause DNA damage and mutations in non-malignant cells (Spanò et al., 2008; Takahashi-Kanemitsu et al., 2020). For example, pks + *Escherichia coli* strains generate the secondary metabolite colibactin, which alkylates and crosslinks DNA bases, leading to double-strand breaks. Mouse models suggest a role for these bacteria in inflammation-related CRC tumorigenesis (Xue et al., 2019). Additionally, toxins from bacteria like *Bacteroides fragilis* enterotoxin (Bft) stimulate the production of reactive oxygen species, resulting in DNA base oxidation and strand breakage, contributing to CRC development (Irrazabal et al., 2020).

**F:** Various bacterial effectors and adhesins can activate cancer-promoting signaling pathways. For instance, the H. pylori cytotoxin-associated gene A (CagA) protein not only promotes hypermethylation of tumor-suppressor genes like CDKN2A but also interacts with E-cadherin, disrupting β-catenin signaling in epithelial cells, thereby contributing to gastric cancer tumorigenesis (Yong et al., 2015). Similarly, the *Fusobacterium nucleatum* adhesin FadA binds to E-cadherin, leading to the translocation of β-catenin into the nucleus, which facilitates the development of CRC (Rubinstein et al., 2013).

The intricate interplay between gut microbiota and tumor dynamics underscores the need for a paradigm shift toward precision microbiome therapeutics. As highlighted in recent bibliometric analyses, emerging research emphasizes the dual role of microbial metabolites—such as SCFAs and tryptophan derivatives—in modulating both pro- and anti-tumor immune responses (He et al., 2021; Tintelnot et al., 2023). For instance, while butyrate enhances CD8 + T cell cytotoxicity, its immunosuppressive effects on dendritic cells during radiotherapy reveal context-specific limitations (Yang et al., 2021). These findings align with broader trends in gastrointestinal oncology, where microbial heterogeneity and host-microbe co-evolution are increasingly recognized as critical determinants of therapeutic outcomes (Wong and Yu, 2023). Future efforts must prioritize multi-omics integration—combining metagenomic, metabolomic, and immune profiling—to identify predictive biomarkers (e.g., *Akkermansia muciniphila* abundance or microbial consortia stability) that guide personalized interventions (Schupack et al., 2022). Additionally, the paradoxical roles of pathobionts like *Fusobacterium nucleatum*—driving tumorigenesis while sensitizing tumors to immunotherapy—call for spatiotemporal mapping of microbial activity within the TME to optimize combinatorial strategies (Gao et al., 2021; Zhao et al., 2023).

### Drug delivery

Bacteria-based cancer therapy represents an innovative approach within synthetic biology, providing promising avenues for cancer treatment (El Tekle and Garrett, 2023). Tumor-targeting bacteria can serve as delivery vectors, enhancing the specificity of drug delivery while minimizing toxicity to patients. These bacteria can effectively reach necrotic or hypoxic regions of tumors, areas often inaccessible to conventional treatments due to compromised tumor vasculature (Zhou et al., 2018).

*E. coli*, *Serratia marcescens*, and *Salmonella Typhimurium* have been extensively studied as biological vehicles for creating biohybrid microswimmers designed to transport larger therapeutic cargoes (Hosseinidoust et al., 2016; Lynch et al., 2022). These microswimmers are self-propelled and equipped with environmental sensing capabilities, making them ideal for active drug transport. For instance, microswimmers formed by attaching drug-loaded microparticles with magnetic nanoparticles to *E. coli* have enabled magnetically guided delivery of doxorubicin to breast cancer cells *in vitro* (Park et al., 2017). In preclinical models of CRC, colonization with engineered *E. coli* Nissle (EcN), which converts ammonia—a metabolic waste product in the tumor microenvironment—into l-arginine, has been shown to enhance T cell infiltration and synergize with anti-PD-L1 treatment (Canale et al., 2021). Engineered strains that express l-arginine, PD-L1, CTLA-4 nanobodies, or STING agonists have demonstrated significant efficacy in inducing immune-mediated tumor control in preclinical studies and are progressing toward clinical trials (Luke et al., 2023).

Further advancements include biohybrid microrobots made from motile *E. coli* carrying magnetic nanoparticles loaded with both photothermal and chemotherapeutic agents. These robots can navigate biological matrices and colonize tumor spheroids under magnetic guidance, releasing drugs in response to near-infrared stimuli. A hybrid control strategy that combines magnetic torque-based navigation with autonomous motility has also improved tumor infiltration by Magnetospirillum magneticum, a magnetotactic bacterium, in spheroid models (Akolpoglu et al., 2022; Alapan et al., 2018).

An alternating magnetic field (AMF) is particularly suitable for manipulating tumor-infiltrating bacteria due to its excellent tissue-penetrating ability and safety profile (Sitti and Wiersma, 2020). *E. coli* MG1655 has been utilized to power biohybrid microswimmers carrying red blood cells loaded with doxorubicin and superparamagnetic iron oxide nanoparticles, enabling magnetic directional control while the bacteria provide propulsion (Alapan et al., 2018). Additionally, researchers have engineered AMF-responsive, tumor-targeting bacteria by conjugating a Fe3O4@lipid nanocomposite to genetically modified *E. coli* BL21, which expresses HlpA—an agent that binds to heparan sulfate proteoglycans commonly overexpressed in CRCs—along with anti-CD47 nanobodies (Ma et al., 2023).

The convergence of synthetic biology and microbiome engineering holds transformative potential for CRC therapy, yet clinical scalability and safety remain paramount challenges. Engineered probiotics, such as *Escherichia coli* Nissle 1917, demonstrate efficacy in preclinical models by reprograming tumor metabolism (Canale et al., 2021), while phage-mediated targeting of dysbiotic pathogens like *Klebsiella pneumoniae* offers precision modulation of gut ecology (Federici et al., 2022). However, as noted in large-scale bibliometric evaluations, the field grapples with standardization gaps—evident in FMT-related risks of pathogen transmission and variable donor efficacy (DeFilipp et al., 2019; Mirzayi et al., 2021). Innovations such as biohybrid microrobots for magnetically guided drug delivery exemplify the promise of interdisciplinary approaches (Ma et al., 2023), yet their translation requires robust validation in human trials. Moving forward, microbiome-informed clinical trials must adopt stratified designs to account for patient-specific microbial signatures, particularly in microsatellite-stable CRC, where immunotherapy resistance persists (Zhao et al., 2023). Furthermore, regulatory frameworks and global collaborations—akin to the STORMS guidelines for microbiome reporting—are essential to harmonize research practices and accelerate therapeutic breakthroughs (Mirzayi et al., 2021).

### Innovative directions and hypotheses

Building on the bibliometric insights, we propose three pioneering hypotheses to address critical gaps in gut microbiota-driven gastrointestinal oncology. First, the “microbial checkpoint” hypothesis posits that specific microbial consortia regulate immunotherapy efficacy by modulating immune checkpoint activity through metabolite-immune crosstalk. For instance, *Akkermansia muciniphila*-enriched microbiomes may enhance PD-1 blockade responses, while *Fusobacterium nucleatum*-dominant ecologies drive resistance—a duality observed in CRC trials (Routy et al., 2018; Zhao et al., 2023). Second, temporal microbiome mapping—tracking microbial dynamics during therapy—could predict and mitigate treatment-induced dysbiosis. Radiotherapy’s dual impact on tumor control and microbiota disruption underscores the urgency of real-time monitoring to optimize intervention timing (Yang et al., 2021). Third, host-microbe co-evolutionary biomarkers, such as genetic variants in butyrate receptors (e.g., GPR109A) or TLR4 polymorphisms, may stratify patients for microbiota-targeted therapies, addressing heterogeneity in treatment outcomes (Schupack et al., 2022).

To translate these concepts, we advocate for translational pipelines integrating multi-omics data with AI-driven predictive models. Machine learning algorithms trained on metagenomic and serum metabolomic profiles could identify microbial signatures predictive of chemotoxicity or immunotherapy resistance, enabling preemptive microbiota modulation. Furthermore, CRISPR-engineered probiotics designed to deliver tumor-suppressive metabolites (e.g., indole-3-carboxaldehyde) or degrade oncogenic toxins (e.g., colibactin) represent an untapped frontier. Coupled with standardized protocols for FMT and microbial consortia administration (Mirzayi et al., 2021), these strategies could redefine adjuvant care in gastrointestinal oncology, particularly for microsatellite-stable tumors resistant to current immunotherapies. By bridging mechanistic insights with synthetic biology, this framework advances beyond descriptive summaries, offering actionable pathways to harness the microbiome’s full therapeutic potential.

## Limitation

This study has several limitations. First, the reliance on English-language articles from the Web of Science Core Collection (WoSCC) may exclude regionally significant innovations published in non-English journals (e.g., Chinese herbal medicine-microbiota interactions), potentially overlooking 8% of relevant studies identified in our preliminary search. Second, citation bias may skew results toward high-impact Western journals (e.g., *Science*, *Nature*), underrepresenting contributions from low- and middle-income countries. Third, the bibliometric methodology inherently carries a temporal lag; citation bursts reflect historical trends, whereas emerging innovations like CRISPR-engineered probiotics or phage therapies may be underrepresented in the 2014–2024 dataset. Finally, while WoSCC was prioritized for its rigorous indexing and citation network tools, its delayed indexing of 2023–2024 publications may marginally affect trend accuracy. Despite these constraints, this analysis provides a robust foundation for understanding global research trajectories, with all methodological trade-offs explicitly documented to guide future replication and extension.

## Conclusion

The bibliometric analysis of publications on the role of gut microbiota in treating gastrointestinal tumors from 2014 to 2024 reveals significant contributions and emerging trends. Notably, substantial economic investment has established China and the United States as the leading countries in research output. Key areas of focus include the mechanisms by which gut microbiota influence systemic immunity, cancer treatment responses, and drug delivery. This study offers a comprehensive roadmap for future research, highlighting the importance of collaboration in advancing this field.

## Data Availability

The original contributions presented in the study are included in the article/supplementary material, further inquiries can be directed to the corresponding author.
